# Age‐related varus progression of femoral bowing and its related factors in the Iwaki cohort study

**DOI:** 10.1002/jeo2.70587

**Published:** 2025-12-07

**Authors:** Takamasa Uehara, Eiji Sasaki, Kyota Ishibashi, Ryo Tomita, Ryoto Kura, Yukiko Sakamoto, Yuka Kimura, Sunao Tanaka, Eiichi Tsuda, Yasuyuki Ishibashi

**Affiliations:** ^1^ Department of Orthopedic Surgery Hirosaki University Graduate School of Medicine Hirosaki Japan; ^2^ Center of Innovation Research Initiatives Organization Hirosaki University Graduate School of Medicine Hirosaki Japan; ^3^ Department of Rehabilitation Medicine Hirosaki University Graduate School of Medicine Hirosaki Japan

**Keywords:** bone metabolism markers, bone mineral density, femoral bowing, knee osteoarthritis

## Abstract

**Purpose:**

To investigate age‐related changes of femoral bowing and its association with bone mineral density (BMD) and bone metabolism using real‐world data from the Iwaki cohort study.

**Methods:**

Data from 709 participants were collected within 10 days in June 2022. The right femoral bowing angle was measured on full‐length standing radiographs of the lower limbs, with varus alignment recorded as positive. BMD was assessed using dual‐energy X‐ray absorptiometry and evaluated based on T‐scores. Bone metabolism was assessed via serum levels of type I procollagen N‐terminal propeptide, type I collagen cross‐linked N‐telopeptide, tartrate‐resistant acid phosphatase 5b and pentosidine. Knee symptoms were evaluated using the Knee Injury and Osteoarthritis Outcome Score. Age‐related changes in femoral bowing angle were analyzed by sex and age group, and regression analysis was performed to identify factors associated with femoral bowing angle.

**Results:**

The mean femoral bowing angle was –1.4 ± 3.8° (31.9% femoral bowing angle showed varus) in male participants and –0.9 ± 3.9° (35.3% femoral bowing angle showed varus) in female participants. In both sexes, femoral bowing angle significantly increased with age, particularly after 60 years. Regression analysis demonstrated that femoral bowing angle was positively associated with age and body mass index (BMI) and bone metabolism markers in female participants. Femoral bowing angle was negatively associated with BMD and grip strength in both sexes. Higher BMI and lower BMD were independently associated with increased femoral bowing angle in both sexes. Femoral bowing angle negatively correlated with the Knee Injury and Osteoarthritis Outcome Score subscale scores.

**Conclusions:**

Femoral lateral bowing progressed towards varus in male and female participants >60 years of age, and was associated with aging, obesity, muscle weakness, reduced bone mineral density and high‐turnover bone metabolism. Knee symptoms were negatively correlated with varus progression of femoral bowing.

**Level of Evidence:**

Level II.

## Graphical abstract

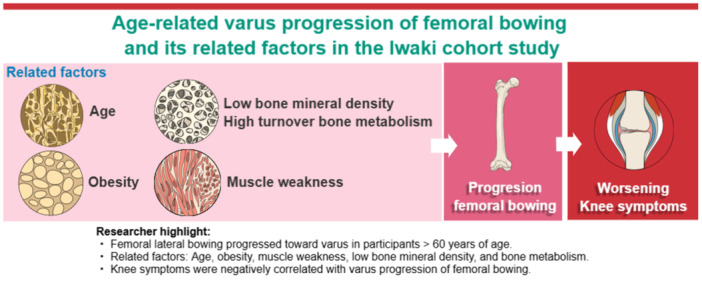

**Abbreviations**
BMD: bone mineral density
BMI: body mass index
FTA: femorotibial angle
HKAA: hip‐knee‐ankle angle
ICCs: intraclass correlation coefficients
JLCA: joint line convergence angle
KL: Kellgren–Lawrence
KOA: knee osteoarthritis
mLDFA: mechanical lateral distal femoral angle
MPTA: medial proximal tibial angle
OA: osteoarthritis
SD: standard deviations
TKA: total knee arthroplasty
WBLR: weight‐bearing line ratio

## INTRODUCTION

Knee osteoarthritis (KOA), a major public health concern among middle‐aged and older individuals, significantly affects activities of daily living (ADL) and quality of life (QOL) [5]. Preventive strategies for KOA help reduce functional impairment and contribute to lowering healthcare costs. Identifying risk factors for KOA progression and implementing appropriate interventions are essential for developing effective prevention strategies [4]. While coronal plane lower limb radiographic parameters primarily focus on deformities around the knee joint, because varus alignment is a key risk factor for KOA progression [27], that may also be associated with femoral deformities. Greater femoral bowing has been suggested to correlate with KOA progression [17, 18, 28].

Lateral femoral bowing is an irreversible morphological change, with osteotomy being the only corrective intervention [23, 25]. Greater lateral femoral bowing is a known contributor to atypical femoral fractures (AFF) [29]. Although often overlooked in the assessment of lower limb alignment during knee arthroplasty or osteotomy procedures, femoral bowing angle (FBA) should be considered a critical parameter in preoperative planning [1]. Thus, preventing the progression of femoral lateral bowing is essential. However, its underlying mechanisms remain unclear. Notably, marked racial differences in femoral lateral bowing prevalence have been observed, with a higher incidence reported in Asian women [1, 14]. Additionally, femoral lateral bowing tends to increase with age [11, 17, 30] and is associated with low bone mineral density (BMD) [9, 28]. In contrast, no significant association has been found between femoral lateral bowing and bone metabolism [30]. Indeed, the mechanisms underlying femoral lateral bowing from a bone metabolism perspective remain largely unknown, and elucidation of their age‐related changes and associated factors could contribute to establish strategies and targets to prevent its progression.

This cross‐sectional study aimed to investigate age‐related changes in femoral lateral bowing and its associated factors, including BMD and bone metabolism, using data from a general population cohort. Also, the difference of lateral femoral bowing between male and female participants was investigated. The hypothesis was that age‐related changes in femoral bowing differ between male and female participants and that femoral lateral bowing is associated with BMD and bone metabolism markers. By clarifying age‐related changes in femoral lateral bowing and their associated factors, this study provides novel insights into the mechanisms underlying femoral bowing development.

## MATERIALS AND METHODS

### Iwaki cohort and patient recruitment

This cross‐sectional study was approved by the Ethics Committee of the Hirosaki University Graduate School of Medicine (Approval Number: 2021‐019H_7) and conducted in accordance with the 1964 Declaration of Helsinki and its subsequent amendments. All participants voluntarily enrolled in the Iwaki Health Promotion Project, a community‐based preventive medicine program aimed at improving life expectancy through health screenings and preventive interventions [3, 12, 21]. Epidemiological studies on knee‐related conditions within the Iwaki Project began in 2008. Written informed consent was obtained from all participants before their inclusion in the study.

**Figure 1:**
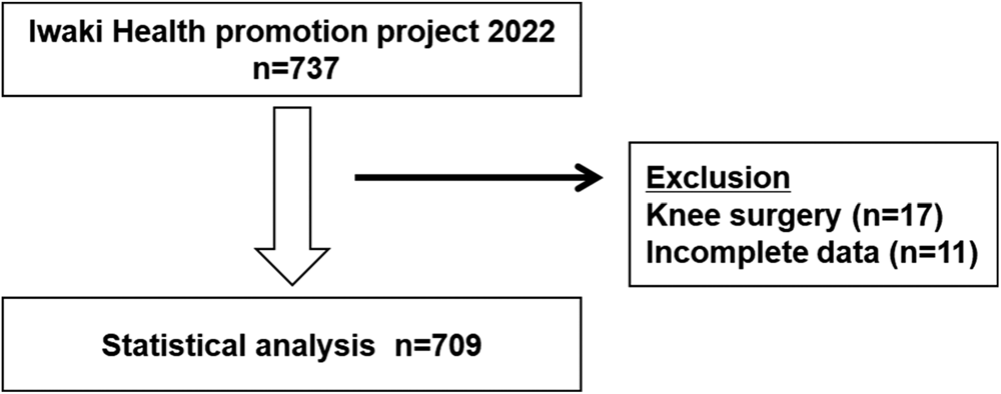
Participants recruitment flow.

In the 2022 Iwaki Health Promotion Project, 737 volunteers (307 male and 430 female participants) were registered (Figure 1). These volunteers were recruited through mass media advertisements and by public health nurses. Participants completed a questionnaire covering their past and current medical history, lifestyle habits, occupation, family history, health‐related QOL and disease‐specific information, including knee symptoms. The following exclusion criteria were applied: 17 participants with a history of knee‐related surgery and 11 with incomplete data. Consequently, 709 individuals (295 male and 414 female participants) were included in the final statistical analysis. Participants were categorized into five age groups: 20–39, 40–49, 50–59, 60–69 and ≥70 years.

### Measurement of radiographic parameters

Knee radiographs were obtained using a digital radiography system (CXDI‐40EG, Canon). Whole‐leg standing radiographs were acquired under the following conditions: source‐to‐image distance, 300 cm; tube voltage, 85 kV; tube current, 200 mA. All participants underwent digital long‐leg radiography while standing barefoot, with their patellae facing forward and their knees fully extended.

**Figure 2:**
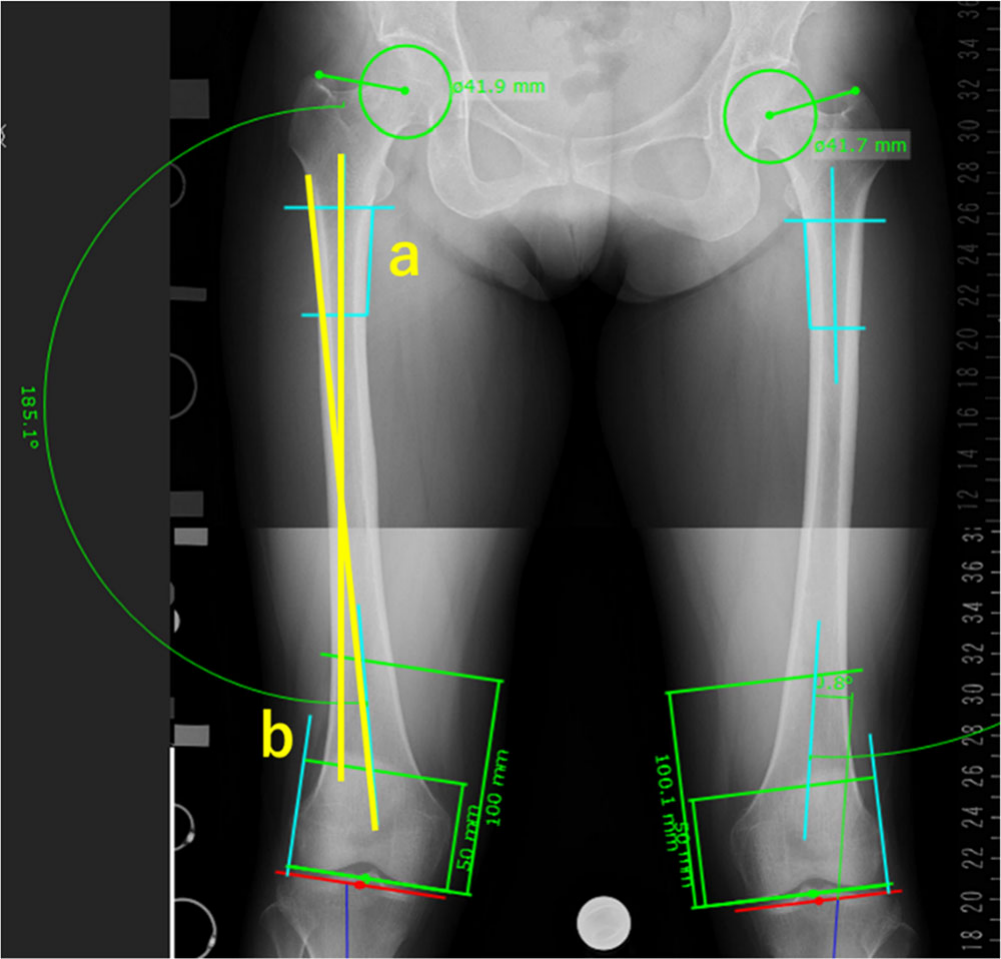
Measurement of FBA. The femoral bowing angle (FBA) is defined as the angle formed by the intersection of the proximal (a) and distal (b) axes. Proximal axis (a): a line connecting the midpoints of the cortical bone, 0 and 5 cm from the lesser trochanter. Distal axis (b): A line connecting the midpoints of the cortical bone at 5 and 10 cm from the distal articular surface of the femur.

Femoral lateral bowing was assessed using the FBA of the right lower limb, measured with mediCAD® software version 5.5 (TOYO Corporation). The FBA was calculated as the angle between the proximal and distal axes [13, 15]. The proximal axis was drawn by connecting the midpoints of the cortical bone at 0 and 5 cm from the lesser trochanter, whereas the distal axis was drawn by connecting the midpoints of the cortical bone at 5 and 10 cm from the distal articular surface of the femur (Figure 2).

If the height of the femoral condyle exceeded 5 cm, the distal axis was defined as the line connecting the midpoints of the cortical bone at 7 and 10 cm from the articular surface of the femur. A positive FBA value indicates varus alignment, whereas a negative value indicates valgus alignment.

Interobserver reliabilities were evaluated using intraclass correlation coefficients (ICCs). For interobserver reliability, 100 FAs of 100 radiographs were independently measured by a senior orthopedic surgeon and a resident orthopedic surgeon. The ICC (2.1) value was 0.994 (95% confidence interval [CI]: 0.991–0.996, *p* < 0.001) for interobserver reliability.

### BMD

BMD of the forearm was measured using dual‐energy X‐ray absorptiometry (DCS‐600EXV; Hitachi Aloka Medical). The measurement site was defined as the distal third of the nondominant forearm. If a participant had a history of fracture in the nondominant forearm, the dominant forearm was measured.

### Blood sample analysis

Fasting blood samples were collected from all participants early in the morning of the day when the bone metabolism markers were measured. Participants were instructed to fast for at least 10 h before blood collection. Blood analyses were outsourced to LSI Medience Corporation, an ISO 15189‐accredited laboratory, which followed rigorous quality control measures. The following bone metabolism markers were analyzed: Bone formation marker: Procollagen type I N‐terminal propeptide (PINP, μg/L, ECLIA method; LSI Medience Corporation) [6], Bone resorption markers: Type I collagen cross‐linked N‐telopeptide (NTx, nM BCE/L, EIA method; LSI Medience Corporation); [10] tartrate‐resistant acid phosphatase‐5b (TRACP‐5b, mU/dL, EIA method; LSI Medience Corporation) [10] and Bone fragility marker: Pentosidine (pmol/mL, HPLC method; LSI Medience Corporation) [26]. These markers are widely used to assess bone turnover and quality in clinical practice. The Japanese Society for Bone and Mineral Research recommends them as clinical indicators for diagnosis and treatment [20]. The intra‐ and interassay coefficients of variation (CVs) for each marker were as follows: 0.9%–1.6% and 1.1%–1.7% (PINP); 6.0%–11.6% and 6.9%–11.1% (NTx); 1.6%–2.9% and 1.8%–7.5% (TRACP‐5b); 2.6%–3.9% and 9.2%–11.1% (pentosidine).

### Knee symptom and strength assessment

Knee symptoms were evaluated using the knee injury and osteoarthritis outcome score (KOOS), a patient‐reported outcome measure. All participants independently completed the KOOS questionnaire, which is a knee‐specific, self‐administered tool comprising 42 items across five subscales: pain, symptoms, ADL, sports and knee‐related QOL. Each item is scored on a scale from 0 to 4, and the total score is converted to a 0–100 scale, where 100 represents the best outcome and 0 represents the worst outcome [19, 24].

To assess skeletal muscle strength, the grip strength was measured twice using a digital measured device, and the average value was recorded for analysis. If only one measurement was available, that value was used (*n* = 2).

### Statistical analysis

To achieve a statistical power of 80% with *α* = 0.05, indicating a large effect size (*r* = 0.5), power analysis determined that 128 participants were required to detect differences in FBA between male and female participants using the Mann–Whitney *U*‐test. In the post hoc analysis, the statistical power for the 709 participants was calculated as 0.999.

Data were analysed using RStudio version 4.2.1 (Posit PBC Inc) and SPSS version 27.0J (SPSS Inc), with a *p*‐value below 0.05 considered statistically significant. Demographic data are presented as mean ± standard deviation (SD). The Shapiro–Wilk test indicated that most demographic parameters and bone metabolism markers did not follow a normal distribution. Consequently, the Mann–Whitney *U*‐test was used to compare demographic data, bone metabolism markers and FBA between male and female participants.

Age‐related differences in FBA were analysed separately for males and females using analysis of variance (ANOVA), followed by Tukey's post hoc test. Spearman′s rank correlation coefficient was used to assess relationships among FBA, age, body mass index (BMI), BMD, KOOS, grip strength and bone metabolism markers. To evaluate the relationship between FBA and BMD, multiple linear regression analysis was conducted with FBA as the dependent variable and age, BMI, BMD and grasping force as independent variables. Additionally, to assess the relationship between bone metabolism markers and FBA, multiple linear regression analysis was conducted with FBA as the dependent variable and age, BMI, BMD, grasping force and bone metabolism markers as independent variables.

## RESULTS

A total of 295 male participants and 414 female participants were enrolled. Male participants had significantly higher BMI and grip strength than female participants, while there was no significant difference in BMD and bone metabolic markers, except for total PINP (Table 1). Regarding BMD and bone metabolism, P1NP levels were significantly higher in female than male participants, while the BMD T‐scores were higher in male than female participants (*p* = 0.075). The mean FBA was −1.4 ± 3.8° in male participants, with 31.9% exhibiting varus femur alignment, whereas in female participants, the mean FBA was −0.9 ± 3.9° (*p* = 0.436), with 35.3% exhibiting varus femur alignment.

**Table 1:** Demographic data of the participants.

	Overall	Male	Female	*p* value
Participants number	709	295	414	—
Age, years	53.2 ± 15.0	54.4 ± 14.8	52.3 ± 15.1	0.068
Body mass index, kg/m²	23.1 ± 3.3	24.0 ± 3.1	22.5 ± 3.4	<0.001
Grip strength, kg	31.2 ± 9.4	40.0 ± 7.5	25.0 ± 4.1	<0.001
Bone mineral density, T‐score, %	96.0 ± 13.3	98.0 ± 9.1	94.5 ± 15.5	0.083
Total P1NP, μg/L	47.0 ± 19.3	44.6 ± 17.7	48.6 ± 20.2	0.013
TRACP‐5b, mU/dL	386.0 ± 180.5	374.0 ± 145.2	394.5 ± 201.7	0.759
NTx, nM BCE/L	17.4 ± 6.7	17.4 ± 7.5	17.5 ± 6.2	0.358
Pentosidine, pmol/mL	25.4 ± 26.3	27.5 ± 39.6	24.0 ± 8.2	0.338
KL grade 0/1, %	65.7	79.7	55.8	—
KL grade 2, %	27.2	14.6	36.2	—
KL grade 3, %	6.8	5.7	7.5	—
KL grade 4, %	0.3	0	0.5	—

*Note*: Mean and standard deviation of age, body mass index, grip force and bone mineral density, total procollagen type I N‐terminal propeptide (PINP), N‐telopeptide NTx), (TRACP‐5b) and Pentosidine between men and women were compared using Mann–Whitney *U*‐test. A *p*‐value below 0.05 was considered significant.

Abbreviation: KL grade, Kellgren‐Lawrence grade.

In male participants, FBA increased towards varus alignment with age (*r* = 0.326, *p* < 0.001). The mean FBA in the 60–69 age group was significantly higher than in the 20–39 age group by 2.37° (*p* = 0.004), while in the >70 age group, it was 3.50° higher than in the 20–39 age group (*p* < 0.001) (Table 2). In female participants, the mean FBA also increased with age (*r* = 0.371, *p* < 0.001) and became positive after 60 years of age. The mean FBA in the 60–69 age group was significantly higher than that in the 20–39 age group by 2.48° (*p* < 0.001), while in the >70 age group, it was higher than that in the 20–39 age group by 4.91° (*p* < 0.001).

**Table 2:** Age‐related change of FBA in males and females.

	20–39	40–49	50–59	60–69	≥70
Male					
Number, people	52	77	42	71	53
FBA, degree	−2.73 ± 3.18	−2.49 ± 3.78	−1.93 ± 4.06	−0.35 ± 3.72*^†^	0.78 ± 3.29*^†‡^
(1st and 3rd quartile), degree	(−4.00 to 0.60)	(−4.4 to −0.1)	(−4.48 to 0.30)	(−2.9 to 2.2)	(−1.2 to 3.1)
(95% confidence interval [CI]), degree	(−3.61 to −1.84)	(−3.34 to −1.63)	(−3.20 to −0.67)	(−1.23 to 0.52)	(−0.13 to 1.68)
Female					
Number, people	101	89	74	92	58
FBA, degree	−2.32 ± 2.95	−1.80 ± 3.25	−2.14 ± 3.02	0.16 ± 3.77*^†‡^	2.59 ± 4.55*^†‡#^
(1st and 3rd quartile), degree	(−4.10 to −0.70)	(−4.10 to 0.70)	(−4.20 to −0.43)	(−2.55 to 3.13)	(−0.48 to 5.48)
(95% CI), degree	(−2.90 to −1.74)	(−2.49 to −1.12)	(−2.84 to −1.44)	(−0.62 to 0.94)	(1.40–3.78)

*Note*: Mean ± standard deviation of right femoral bowing angle (FBA) was compared using analysis of covariance, and Tukey test as a post hoc. analysis. A *p*‐value below 0.05 was considered significant in comparison with 20–39 age group (*), 40–49 age group (†), 50–59 age group (‡) and 60–69 age group (#).

The mean KOOS scores among all participants were 92.9 ± 13.1 (range: 36.1–100) for pain, 91.2 ± 12.8 (range: 17.9–100) for symptoms, 96.5 ± 8.9 (range: 33.8–100) for ADL, 90.3 ± 19.1 (range: 5.0–100) for sports/recreation, and 84.9 ± 20.4 (range: 0–100) for QOL. In female participants, FBA was negatively correlated with all five subscales of KOOS (range of correlation coefficients: −0.194 to −0.254) (Table 3).

**Table 3:** Correlation between FBA and knee symptoms.

	Male			Female		
	Mean ± SD		*p* value	Mean ± SD	*r*	*p* value
KOOS symptoms	93.1 ± 10.3	−0.028	0.630	89.8 ± 14.2	−0.194	<0.001
KOOS pain	94.6 ± 11.1	−0.111	0.056	91.7 ± 14.2	−0.213	<0.001
KOOS ADL	97.3 ± 7.8	−0.192	<0.001	95.9 ± 9.6	−0.224	<0.001
KOOS sports/recreation	92.6 ± 15.8	−0.193	<0.001	88.5 ± 21.3	−0.254	<0.001
KOOS QOL	88.6 ± 17.2	−0.141	0.015	82.3 ± 21.9	−0.248	<0.001

*Note*: Mean ± standard deviations (SD) of knee injury and osteoarthritis outcome scales (KOOS) subscales in males and females were shown. Correlations between femoral bowing angle (FBA) and KOOS subscales were estimated. *p* < 0.05 was considered significant.

Abbreviations: ADL, activities of daily living; QOL, quality of life.

Crude linear regression analysis showed that in male participants, FBA was positively correlated with age and negatively correlated with the BMD T‐score and grip strength. In female participants, FBA was positively correlated with age, BMI, total P1NP, TRACP‐5b, serum NTx and pentosidine while negatively correlated with the BMD T‐score and grip strength (Table 4). Furthermore, multiple linear regression analysis confirmed significant associations of FBA with higher BMI and lower BMD in male and female participants.

**Table 4:** Related factors for FBA.

	Male				Female			
	Crude		Adjusted		Crude		Adjusted	
	*β*	*p* value	*β*	*p* value	*β*	*p*‐Value	*β*	*p* value
Age	0.33	<0.001	0.26	<0.001	0.48	<0.001	0.11	0.134
BMI	0.11	0.053	0.19	0.002	0.27	<0.001	0.25	<0.001
BMD T‐score	−0.26	<0.001	−0.16	0.011	−0.42	<0.001	−0.28	<0.001
Grip strength	−0.18	0.002	−0.08	0.196	−0.12	0.019	0.01	0.848
Total P1NP	−0.21	0.717	0.03	0.727	0.10	0.043	−0.03	0.668
TRACP‐5b	0.11	0.059	0.04	0.579	0.26	<0.001	0.01	0.895
Serum NTx	0.01	0.908	0.05	0.381	0.16	0.001	0.03	0.654
Pentosidine	0.03	0.629	−0.08	0.256	0.21	<0.001	0.09	0.081

*Note*: Statistical analysis—linear regression analysis. Dependent variable: FBA. independent variables—age, sex, BMI, BMD, grasping force, total P1NP, TRACP‐5b, NTx, Pentosidine, *β*: coefficients.

Abbreviations: BMD, body mineral density; BMI, body mass index; FBA, femoral bowing angle; NTx, N‐telopeptide; total P1NP, procollagen type I N‐terminal propeptide.

## DISCUSSION

This large‐sample cohort study revealed age‐related changes in femoral lateral bowing in the general population, identifying 60–69 years as the critical decade for increasing FBA towards varus alignment compared with younger individuals in male and female participants. Mean FBA in female participants was significantly higher than that of male participants. Factors associated with FBA include age, obesity, sarcopenia, lower BMD and high bone turnover, particularly in women.

This study demonstrated that FBA significantly increased with age in both male and female participants aged ≥60 years. Additionally, the magnitude of age‐related FBA changes was greater in female participants than in male participants. Previous Korean studies have also reported a notable increase in FBA in female participants after the age of 6.0 while those of male participants have showed mild gradual increases. The 60–70 age group (4.14 ± 5.44°) exhibited an approximately 5.71° greater FBA than the 20–30 age group (−1.57 ± 2.64°), and a significant increase was observed when comparing the 50–60 age group (0.21 ± 4.22°) with those aged ≥60 years [14]. Therefore, the marked increase in FBA after age 60 observed in this study aligns with previous findings, reinforcing these observations. Based on these results, preventive interventions for FBA should be initiated before the age of 60.

FBA changes were predominantly observed in female participants >60 years, which may reflect the effects of menopause‐related estrogen decline and the subsequent high turnover of bone metabolism, while there were few reports about the association between male FBA and hormonal condition. Previous studies have suggested a strong association between femoral lateral bowing, postmenopausal bone density loss and hormonal changes [2, 9, 28]. Following menopause, estrogen levels decline sharply, leading to increased bone resorption and turnover, resulting in a significant reduction in BMD [10, 14]. This explains the higher prevalence of osteoporosis in postmenopausal female participants [8]. Reduced BMD weakens bone rigidity, and prolonged mechanical loading may contribute to progressive femoral bowing [9]. Furthermore, femoral bowing has been associated with osteoporosis, increased fracture risk, atypical femoral fractures and 25‐hydroxyvitamin D (25(OH)D) [22, 29, 30]. The marked increase in FBA in female participants >60 years observed in this study supports the hypothesis that postmenopausal metabolic changes and BMD reduction are key contributors to femoral bowing progression.

In this study, higher BMI and lower grip strength were significantly correlated with FBA, indicating that obesity and muscle weakness may accelerate FBA progression. Grip strength is recognized as an important indicator of whole body muscle strength [31]. These findings are consistent with previous studies that have reported an association between higher BMI and femoral bowing [7, 9]. However, no prior studies have directly linked femoral lateral bowing to muscle mass. KOA is also associated with lower‐limb muscle weakness has been reported in older female participants with KOA [16, 32].

Finally, a significant negative correlation was found between FBA and knee symptoms, as assessed using the KOOS, a validated measure for quantifying knee‐related symptoms [19, 24]. These findings suggest that increased FBA is associated with worsening knee symptoms. These results imply that FBA progression, a known risk factor for KOA, contributes to the deterioration of knee symptoms.

This study has some limitations. First, BMD was measured at the forearm. Although hip and spinal BMD generally provide a more accurate reflection of overall bone health, their assessment requires radiation‐shielded facilities. As this study was conducted as part of a community health initiative, forearm BMD assessment was performed due to space constraints at the community center. Second, grip strength was used as an indicator of muscle mass. Although lower limb muscle strength is generally preferred for evaluating its relationship with KOA, this study utilized a dataset from a large‐scale health screening program, necessitating the use of grip strength as a more practical assessment method. Third, as this was a cross‐sectional study, causal relationships between femoral bowing and variables such as age, BMI and BMD could not be established. Finally, in large‐scale population‐based studies, effect sizes must be considered, and careful interpretation of results is necessary. Therefore, these findings may not be generalizable to broader populations. Despite these limitations, this study provides valuable insights into the mechanisms underlying femoral bowing and serves as a foundation for future research.

## CONCLUSIONS

Femoral lateral bowing increased towards varus more in female participants after age 60. Also, increasing femoral lateral bowing was associated with age, higher BMI, weaker grip strength, lower BMD and high‐turnover bone metabolism as underlying mechanisms. Furthermore, knee symptoms were negatively correlated with FBA.

## AUTHOR CONTRIBUTIONS

Eiji Sasaki was responsible for the organization and coordination of this study and was the chief investigator responsible for data analysis. Takamasa Uehara also contributed in writing and analyzing for this study. Kyota Ishibashi, Ryo Tomita, Ryoto Kura, Yukiko Sakamoto, Yuka Kimura, Sunao Tanaka, Eiichi Tsuda and Yasuyuki Ishibashi developed the study design and advised for the analysis and data interpretation. All authors contributed to the writing of the final manuscript.

## CONFLICT OF INTEREST STATEMENT

The authors declare no conflicts of interest.

## ETHICS STATEMENT

The study was approved by the Ethics Committee of Hirosaki University Graduate School of Medicine (No. 2021‐019H_7) and was conducted in accordance with the 1964 Helsinki Declaration and its later amendments or comparable ethical standards. All participants provided written informed consent before participation.

## Data Availability

The datasets generated and/or analyzed in the current study are available from the corresponding author upon reasonable request.

## References

[1] Abdelaal AHK , Yamamoto N , Hayashi K , Takeuchi A , Morsy AF , Miwa S , et al. Radiological assessment of the femoral bowing in Japanese population. SICOT‐J. 2016;2:2. 27163091 10.1051/sicotj/2015037 PMC4849268

[2] Ahlborg HG , Johnell O , Turner CH , Rannevik G , Karlsson MK . Bone loss and bone size after menopause. N Engl J Med. 2003;349:327–334. 12878739 10.1056/NEJMoa022464

[3] Chiba D , Maeda S , Sasaki E , Ota S , Nakaji S , Tsuda E , et al. Meniscal extrusion seen on ultrasonography affects the development of radiographic knee osteoarthritis: a 3‐year prospective cohort study. Clin Rheumatol. 2017;36:2557–2564. 28920170 10.1007/s10067-017-3803-6

[4] Cooper C , Snow S , McAlindon TE , Kellingray S , Stuart B , Coggon D , et al. Risk factors for the incidence and progression of radiographic knee osteoarthritis. Arthritis Rheum. 2000;43:995–1000. 10817551 10.1002/1529-0131(200005)43:5<995::AID-ANR6>3.0.CO;2-1

[5] Cross M , Smith E , Hoy D , Nolte S , Ackerman I , Fransen M , et al. The global burden of hip and knee osteoarthritis: estimates from the global burden of disease 2010 study. Ann Rheum Dis. 2014;73:1323–1330. 24553908 10.1136/annrheumdis-2013-204763

[6] Delmas PD , Eastell R , Garnero P , Seibel MJ , Stepan J , Committee of Scientific Advisors of the International Osteoporosis Foundation . The use of biochemical markers of bone turnover in osteoporosis. Osteoporos Int. 2000;11:S2–S17. 11193237 10.1007/s001980070002

[7] Do S , Lee CG , Kim DH , Lee G , Kim KY , Ryu SY , et al. Factors related to femoral bowing among Korean female farmers: a cross‐sectional study. Ann Occup Environ Med. 2020;32:e23. 32802339 10.35371/aoem.2020.32.e23 PMC7406743

[8] Faienza MF , Ventura A , Marzano F , Cavallo L . Postmenopausal osteoporosis: the role of immune system cells. Clin Dev Immunol. 2013;2013:575936. 23762093 10.1155/2013/575936 PMC3677008

[9] Furihata Y , Ishikawa T , Katsuragi J , Omae T , Sasaki Y , Umimura T , et al. Lateral bowing of femur associated with older age, shorter stature, and lower bone mineral density. Cureus. 2021;13:e19735. 34950542 10.7759/cureus.19735 PMC8687800

[10] Garnero P . Markers of bone turnover in prostate cancer. Cancer Treat Rev. 2001;27:187–192. 11417970 10.1053/ctrv.2000.0213

[11] Hwang D , Wook Choi M , Kim SH , Han HS , Bum Chang C , Chul Lee M , et al. Age and sex differences in coronal lower extremity alignment in a healthy Asian population. Knee. 2023;45:198–206. 37931367 10.1016/j.knee.2023.09.009

[12] Ishibashi K , Sasaki E , Ota S , Oyama T , Chiba D , Yamamoto Y , et al. Bone marrow lesion severity was associated with proximal tibial inclination in early knee osteoarthritis. Knee Surg Sports Traumatol Arthrosc. 2022;30:668–679. 33394079 10.1007/s00167-020-06378-7

[13] Kim JM , Hong SH , Kim JM , Lee BS , Kim DE , Kim KA , et al. Femoral shaft bowing in the coronal plane has more significant effect on the coronal alignment of TKA than proximal or distal variations of femoral shape. Knee Surg Sports Traumatol Arthrosc. 2015;23:1936–1942. 24760162 10.1007/s00167-014-3006-5

[14] Kim JY , Kong GM . Age‐ and gender‐related femoral bowing analysis in the korean population and features for clinical applications. Medicina. 2024;60:1930. 39768812 10.3390/medicina60121930 PMC11677447

[15] Lasam MPG , Lee KJ , Chang CB , Kang YG , Kim TK . Femoral lateral bowing and varus condylar orientation are prevalent and affect axial alignment of TKA in Koreans. Clin Orthop Rel Res. 2013;471:1472–1483. 10.1007/s11999-012-2618-7 PMC3613555 23011845

[16] Lee S , Kim TN , Kim SH . Sarcopenic obesity is more closely associated with knee osteoarthritis than is nonsarcopenic obesity: a cross‐sectional study. Arthr Rheum. 2012;64:3947–3954. 23192792 10.1002/art.37696

[17] Matsumoto T , Hashimura M , Takayama K , Ishida K , Kawakami Y , Matsuzaki T , et al. A radiographic analysis of alignment of the lower extremities‐‐initiation and progression of varus‐type knee osteoarthritis. Osteoarthr Cartilage. 2015;23:217–223. 10.1016/j.joca.2014.11.015 25481289

[18] Mullaji AB , Marawar SV , Mittal V . A comparison of coronal plane axial femoral relationships in Asian patients with varus osteoarthritic knees and healthy knees. J Arthroplasty. 2009;24:861–867. 18701244 10.1016/j.arth.2008.05.025

[19] Nakamura N , Takeuchi R , Ishikawa H , Saito T , Sawaguchi T , Goldhahn S . Cross‐cultural adaptation and validation of the Japanese Knee Injury and Osteoarthritis Outcome Score (KOOS). J Orthop Sci. 2011;16:516–523. 21766211 10.1007/s00776-011-0112-9

[20] Nishizawa Y , Ohta H , Miura M , Inaba M , Ichimura S , Shiraki M , et al. Guidelines for the use of bone metabolic markers in the diagnosis and treatment of osteoporosis (2012 edition). J Bone Miner Metab. 2013;31:1–15. 23143508 10.1007/s00774-012-0392-y

[21] Ota S , Chiba D , Sasaki E , Kumagai G , Yamamoto Y , Nakaji S , et al. Symptomatic bone marrow lesions induced by reduced bone mineral density in middle‐aged women: a cross‐sectional Japanese population study. Arthritis Res Ther. 2019;21:113. 31060629 10.1186/s13075-019-1900-4 PMC6501306

[22] Papaioannou I , Pantazidou G , Baikousis A , Korovessis P . Femoral bowing and femoral neck‐shaft angle evaluation can reduce atypical femoral fractures in osteoporotic patients: a scientific report. Cureus. 2020;12:10771. 10.7759/cureus.10771 PMC7606192 33154843

[23] Peng H , Ou A , Huang X , Wang C , Wang L , Yu T , et al. Osteotomy around the knee: the surgical treatment of osteoarthritis. Orthop Surg. 2021;13:1465–1473. 34110088 10.1111/os.13021 PMC8313165

[24] Roos EM , Roos HP , Lohmander LS , Ekdahl C , Beynnon BD . Knee injury and osteoarthritis outcome score (KOOS)‐‐development of a self‐administered outcome measure. J Orthop Sports Phys Ther. 1998;28:88–96. 9699158 10.2519/jospt.1998.28.2.88

[25] Rossi R , Cottino U , Bruzzone M , Dettoni F , Bonasia DE , Rosso F . Total knee arthroplasty in the varus knee: tips and tricks. Int Orthop. 2019;43:151–158. 30141140 10.1007/s00264-018-4116-3

[26] Saito M , Marumo K . Collagen cross‐links as a determinant of bone quality: a possible explanation for bone fragility in aging, osteoporosis, and diabetes mellitus. Osteoporos Int. 2010;21:195–214. 19760059 10.1007/s00198-009-1066-z

[27] Sharma L , Song J , Dunlop D , Felson D , Lewis CE , Segal N , et al. Varus and valgus alignment and incident and progressive knee osteoarthritis. Ann Rheum Dis. 2010;69:1940–1945. 20511608 10.1136/ard.2010.129742 PMC2994600

[28] Shimosawa H , Nagura T , Harato K , Kobayashi S , Nakamura M , Matsumoto M , et al. Variation of three‐dimensional femoral bowing and its relation to physical status and bone mineral density: a study with CT. Surg Radiol Anat. 2019;41:1489–1495. 31520108 10.1007/s00276-019-02323-7

[29] Soh HH , Chua ITH , Kwek EBK . Atypical fractures of the femur: effect of anterolateral bowing of the femur on fracture location. Arch Orthop Trauma Surg. 2015;135:1485–1490. 26286640 10.1007/s00402-015-2297-4

[30] Tsuchie H , Miyakoshi N , Kasukawa Y , Senma S , Narita Y , Miyamoto S , et al. Factors related to curved femur in elderly Japanese women. Ups J Med Sci. 2016;121:170–173. 27228191 10.1080/03009734.2016.1185200 PMC4967262

[31] Vaishya R , Misra A , Vaish A , Ursino N , D'Ambrosi R . Hand grip strength as a proposed new vital sign of health: a narrative review of evidences. J Health Popul Nutr. 2024;43:7. 38195493 10.1186/s41043-024-00500-y PMC10777545

[32] Zhang X , Pan X , Deng L , Fu W . Relationship between knee muscle strength and fat/muscle mass in elderly women with knee osteoarthritis based on dual‐energy x‐ray absorptiometry. Int J Environ Res Public Health. 2020;17:573. 31963156 10.3390/ijerph17020573 PMC7013934

